# Expression Marker-Based Strategy to Improve Beef Quality

**DOI:** 10.1155/2016/2185323

**Published:** 2016-03-15

**Authors:** Isabelle Cassar-Malek, Brigitte Picard

**Affiliations:** ^1^INRA, UMR1213 Herbivores, 63122 Saint-Genès-Champanelle, France; ^2^Clermont Université, VetAgro Sup, UMR1213 Herbivores, Clermont-Ferrand, 63370 Lempdes, France

## Abstract

For beef cattle research, a main objective is to control concomitantly the development of muscles and the qualities of beef cuts. Beef quality is a complex phenotype that is only detectable after slaughter and is highly variable. The beef industry is in need of tools to estimate beef quality of live cattle or online in abattoirs, with specific attention towards sensory attributes (tenderness, juiciness, flavour, and colour). Identification of relevant genetic and genomic markers is ongoing, especially for tenderness—a top priority quality attribute. In this paper, we describe the steps of an expression marker-based strategy to improve beef sensory quality, from the discovery of biomarkers that identify consistent beef and the biological functions governing beef tenderness to the integration of the knowledge into detection tests for desirable animals. These tools should soon be available for the management of sensory quality in the beef production chain for meeting market's demands and assuring good quality standards.

## 1. Introduction

Ruminant production by providing milk and meat products is a key contributor to protein supply and food security for human beings in many areas of the world. Controlling the performance of ruminants and the quality of their products is therefore an economic challenge. With regard to cattle, the reproduction and fertility of dairy or suckling cows, the nutritional (protein and lipid composition) and sensory quality of milk or meat products, and the environmental footprint (including greenhouse gas emissions and nitrogen excretion) are important issues to be solved. For beef cattle, the main objective is to control concomitantly feed efficiency, development of muscles, and the sanitary, nutritional, and sensory qualities of meat and carcass. However, a serious bottleneck is our poor understanding of the mechanisms that underlie these complex phenotypes. Moreover, there is yet no evaluation system to predict the qualities of beef before slaughtering, except empirical farmers' expertise. The Meat Standard Australian (MSA) system tested by several countries allows a classification of meat cuts only after slaughter [1]. One limit of the MSA system is that the evaluation of meat quality is independent of the biochemistry of the muscles, and it is not operational yet on pure breeds. The phenotypic attributes of beef production need to be depicted in order to predict and manage meat production from live animal to carcass. Expressional genomics of tissues and fluids attempts to link the genome expression and these phenotypic traits including development and quality of tissues, efficiency, and adaptation to nutritional or climatic disturbances. The objective is to propose innovative solutions based on biomarkers for prediction and phenotyping purposes to stakeholders of the beef sector for better allocation of carcasses or cuts to the appropriate markets. In the present paper, we will describe the steps of an expression marker-based strategy to improve beef sensory quality.

## 2. Expression Markers for a Complex Phenotypic Trait

**Figure 1:**
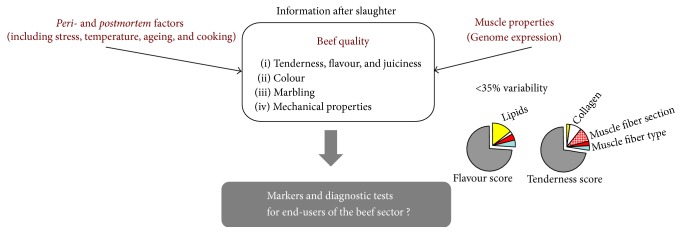
Beef sensory quality. A complex phenotypic trait that is expressed* postmortem*. A combination of molecular processes in the muscle both in the live animal and during the* peri*- and* postmortem* period (stress, interaction of the whole carcass, and muscle characteristics with cooler temperatures affecting rates and extents of* postmortem* pH decline, ageing, and cooking) contributes to development of beef quality. In particular, the muscle characteristics of the live animals play an important role. However, less than one-third to a fourth of the variability in beef tenderness and flavour can be explained by variability in the muscle characteristics of live animals. The beef industry is looking for biological or molecular indicators that would identify live animals with desirable quality attributes, in order to orientate them towards the most accurate production or market system, provided that slaughtering conditions are controlled.

Beef quality is defined by its compositional quality (lean to fat ratio)—determining its nutritional quality—and the palatability factors including appearance (colour, freshness, and marbling), odour, juiciness, tenderness, and flavour—determining its sensory quality. The sensory quality is a complex function of production, processing and meat preparation, and consumer perception. A combination of molecular processes in the muscle of the live animal and during the* postmortem* period contributes to beef quality [2, 3]. Sensory quality is a complex phenotype (Figure 1) that is influenced by intrinsic (e.g., muscle structure, biochemical composition, and* postmortem* changes in muscle tissues) and extrinsic factors (e.g., rearing conditions, stress and preslaughter effects, product handling, chemical environment, processing, and storage) and still needs to be unravelled.

Today, the information on quality is only obtainable after slaughter and ageing (up to 3 weeks) which is a limitation to the delivery of consistent beef quality. More specifically, consumers seek for beef regularity especially for colour—a major criterion at the point of sale— and tenderness—a top priority attribute with high variability [4–6]. However, in Europe beef variability reaches about 20% [4] which is not acceptable. For tenderness, the characteristics of cattle muscles (including fiber type, collagen, and intramuscular lipids) only explain up to 30% of the variability [7]. Variability originates from genetic polymorphisms (reviewed in Picard et al. [8]), modulation of gene expression according to rearing conditions and environment, or uncontrolled* postmortem* processes. Providing consistent beef sensory quality is therefore a critical issue for the beef industry. The beef sector is expecting tools to estimate the “beef potential” of live cattle on the farm or carcasses online.

The advent of genomics has enabled genotyping of animals (using high-density panels of molecular markers) and expression profiling of the entire genome (using microarrays or RNAseq tools and proteomics) in order to identify markers of beef quality. These markers include patterns of SNP, DNA methylation, mRNA, protein, or metabolite expression as long as their pattern can be shown to correlate with the phenotypic expression (e.g., muscle growth, muscle composition, and meat quality attributes) (for a review see [8]). Expressional genomics has allowed the detection of transcripts or proteins differentially expressed or coexpressed, of which the abundance was linked to the development of muscle tissue quantitatively (meat yield) or qualitatively (desirable meat quality) [9–16]. Transcripts and proteins can be considered as expression markers or biomarkers. The final objective is to get information for unravelling the quality attributes, to compute marker abundance in prediction equations, and to predict the “beef potential” of an animal at different times of its life (see Box 1). This strategy can also enable evaluating the impact of nutrition and cattle management practices on the development of tissues and the composition and quality of their food products. To that extent, expression markers may be informative of the interactions between genetic and environmental factors in the construction of beef quality phenotype.

### 2.1. Biomarkers of Beef Tenderness

Tenderness is the beef quality trait most studied during the last decade. Several studies have analysed the evolution of protein profiles in* postmortem* muscles in order to identify biomarkers related to meat conversion [18–20]. Jia et al. [18] studied the proteomic profiles of two bovine muscles differing in their tenderness, the* longissimus thoracis* (LT, tender), and* semitendinosus* (ST, tough) at slaughter and 24 hours later. In both muscle types, they observed a decrease in levels of cofilin (known to promote actin polymerisation), Hsp27, and Hsp20. They distinguished several Hsp27 isoforms as also detected by Chaze et al. [21]. The expression of one isoform dropped dramatically in the LT muscle* postmortem*. Differences were also shown between the two muscles in the* postmortem* profile of proteins involved in glycolytic pathways such as lactoylglutathione lyase (increasing in ST) or triose-phosphate isomerase (dropping in LT). These enzymes reflect the transition to anaerobic metabolism after slaughter with different speed according to the muscle. The authors concluded that the different degrees of tenderness of the two muscles originated from distinct and similar factors associated with physiological, metabolic, and protein differences in two different muscle types. Sawdy et al. [20] analysed the proteomic profiling (2-DE gels and mass spectrometry) of the LT muscle by 36 hours* postmortem*. They identified fragments of the contractile protein myosin heavy chain considered to be a good indicator of tenderness of meat after 7 days of ageing. They proposed these indicators for the classification of carcasses according to their degree of tenderness.

Other studies were conducted in muscle early after death in order to depict molecular profiles at the time of slaughter that could help predict meat tenderness after ageing. Several biomarkers were identified by transcriptomic and proteomic approaches (for review [8]). The biomarkers identified so far belong to several molecular processes of muscle energy metabolism (glycolytic and oxidative), calcium metabolism, ultrastructure and contraction, oxidative stress, apoptosis, and cell protection with a special focus on heat shock proteins (Hsps) (Table 1). According to Juárez et al. [22], 60% of the variability in tenderness is due to ageing hence perhaps explaining the relationships of biomarkers such as Hsp, apoptosis proteins to tenderness. Similarly, prooxidants have the potential to negatively affect meat tenderness by stimulating protein aggregation as well as inhibiting protein degradation [23] possibly explaining the relationship of antioxidant enzymes to tenderness. Interestingly some transcriptional markers related to tenderness were distinct from those related to selection on muscle hypertrophy [24, 25] suggesting that selection for muscle mass would not alter significantly the quality of the meat. However, the combined results of Bouley et al. on markers of tenderness [26] and on markers of muscle hypertrophy [25] also revealed some proteins associated with both tenderness and muscle mass. For example, phosphorylated myosin light chain 2 (MLC2-P), parvalbumin, and myosin binding protein-H (MYBP-H) were positive markers of muscle mass and of tenderness. This indicated that some biomarkers could be useful for the prediction of both muscle hypertrophy and tenderness.

### 2.2. Biomarkers of Juiciness, Flavour, and Colour

**Figure 2:**
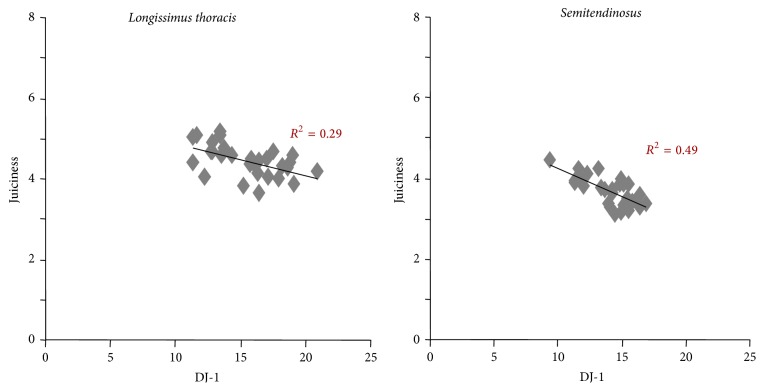
Relationships between the abundance of DJ-1 protein evaluated by dot-blot (arbitrary unit) and the juiciness score estimated by trained panellists on* semitendinosus* and* longissimus thoracis* muscles of Salers young bulls.

Bernard et al. [9] by transcriptomic analysis revealed biomarkers of sensory attributes including juiciness and flavour in Charolais young bulls (Table 2). They found 16 and 17 transcripts positively correlated with flavour and juiciness, respectively, and one negatively correlated with both juiciness and flavour. Hsp40 was correlated (negatively) with tenderness only (Table 2). Many of the candidate biomarkers were common between juiciness and flavour, except for laminin, which was specific for flavour, and protein kinase, AMP-activated, and *γ* 1 noncatalytic subunit (PRKAG1) which was specific for juiciness. Several transcripts including carbonyl reductase 2, hypothetical protein FLJ12193, nucleophosmin/nucleoplasmin 3, and tripartite motif-containing 55 were positively correlated with three sensory attributes (Table 2). These data demonstrate that some biomarkers can be used for the prediction of several sensory qualities. Picard et al. [27] showed by proteomic analysis that several proteins identified as biomarkers of tenderness were also correlated with flavour and/or juiciness in the ST and LT from young bulls of the Salers hardy beef breed. For example, DJ-1 and Hsp 70-GRP75 were negatively correlated with juiciness in both muscles. A multiple regression analysis demonstrated that the protein DJ-1 explained alone 29 % of the juiciness variability in the LT muscle and 49% in the ST muscle (Figure 2). The DJ-1 protein has an antiapoptotic function and a protective activity against oxidative stress. Guillemin et al. [28] showed that DJ-1 interacts with proteins of the small Hsp family through Hsp27. Chelh et al. [29] showed an overexpression of DJ-1 in double-muscled cattle. So this protein is positively associated with muscle mass and negatively with juiciness. However, understanding the involvement of this protein in the juiciness of the meat will need further analysis.

Some of the biomarkers of tenderness were shown to be associated with pH decline and meat colour. For example, peroxiredoxin-6 (PRDX6) abundance was negatively correlated with pH (45 min, 3 h, and pHu) [30] in the LT muscle of French Blond Aquitaine young bulls. The authors also examined the relationships of protein biomarkers of tenderness to CIE-L^*∗*^a^*∗*^b^*∗*^ colour traits. They found that Hsp70-1A/B and *µ*-calpain were correlated with the three colour parameters [30]. These relationships could be explained by the protective role of Hsp70-1A/B on the proteolysis of structural proteins by *µ*-calpain. Other Hsps were correlated with colour attributes. Hsp70-8 and *α*B crystallin (CRYAB) were negatively and positively correlated with L^*∗*^ and b^*∗*^, respectively. A positive correlation between Hsp40 and a^*∗*^ was also detected [30]. This colour attribute was also positively correlated with PRDX6. Some correlations were also found between glycolytic enzymes such as malate dehydrogenase 1 (MDH1), Enolase 3 (ENO3), lactate dehydrogenase B (LDH-B), and pH decline and colour. These data revealed common biomarkers between several quality attributes such as pH decline, colour, and tenderness. However, the mechanisms involving these proteins are different according to the quality attribute.

## 3. Complexity of the Landscape of Biomarkers of Tenderness

### 3.1. Biomarker Abundance according to Intrinsic and Extrinsic Factors

#### 3.1.1. Muscle Type

According to Guillemin et al. [11], the LT muscle exhibits significantly higher abundance of CRYAB, Hsp40, Hsp70-1A/B, and Hsp70-8 than the ST muscle. Abundance of MYBP-H, Myosin heavy chain- (MYHC-) I, phosphoglucomutase (PGM) was also significantly higher in LT than in ST. The ST muscle exhibits significantly higher abundance of Enolase 1 (ENO1) and MYHC-IIx than the LT. A higher expression of PRDX6 is detected in the ST muscle suggesting that the chaperone and antistress activities are lower in a glycolytic muscle. Therefore, PRDX6 could reduce the oxidative injuries by heat shock proteins on unprotected proteins in this muscle. No significant muscle effect was detected for Hsp70/GRP75, ENO3, LDH-B, MDH1, CapZ-*β*, desmin and myosin light chain-1F (MLC-1F), and superoxide dismutase 1 (SOD1). Cassar-Malek et al. [31] showed (at mRNA and protein levels) a higher expression of Hsp40 in oxidative muscles of cattle as observed [17] in LT comparatively to ST muscle. Altogether, these data demonstrate that animal and muscle types are characterised mainly by a differential expression of several Hsp and oxidative resistance proteins, depending on the metabolic and contractile muscle type. The small Hsp family is overexpressed in the LT muscle, more oxidative, to protect proteins against Reactive Oxygen Species (ROS) as also Hsp70-1A and Hsp70-8. In the ST (glycolytic type), we hypothesise that, in case of cellular stress, a strong and active pathway is activated to protect cells against ROS through PRDX6. Indeed, proteins involved in oxidative stress such as PRDX6 were proposed as negative biomarkers of tenderness mainly in glycolytic muscle types [17]. Antioxidant proteins could have a role in the protection of structural proteins against oxidative stress and proteolysis in a muscle type dependent manner.

#### 3.1.2. Animal Type

Guillemin et al. [11] studied the effect of muscle type (LT versus ST) and sex (young bulls versus steers) for 24 biomarkers of tenderness. They showed a significant effect for some Hsp proteins. For example, CRYAB and Hsp27 were significantly more abundant in young bulls than in steers. Conversely, Hsp70-8 and Hsp70/GRP75 were significantly overabundant in steers than in young bulls. Abundance of Hsp40 and Hsp70-1A/B showed no differences between the animal types. The two isoforms of the glycolytic enzyme Enolase (ENO1 and ENO3) were significantly more abundant in steers than in young bulls. Accordingly, abundance of MLC-1F and MYHC-IIx was significantly higher in steers than in young bulls. On the contrary, the muscles of young bulls exhibited significantly higher abundance of MYBP-H than those of steers. Abundance of the antioxidant enzymes DJ-1 and PRDX6 was significantly higher in steers than in young bulls. PRDX6 was significantly different in ST muscle only. However, abundance of SOD1 was not different between animal types and between muscles. The two proteolytic enzymes m-calpain and *μ*-calpain were significantly higher in steers than in young bulls. All these differences in biomarker abundance could explain the weak differences between steers and young bulls observed by several authors [32, 33].

#### 3.1.3. Stress

The relationship of stress to tenderness is well understood in meat science. It is explained by higher depletion of glycogen before slaughter, less production of lactic acid, a by-product of* postmortem* glycolysis, and thus insufficient pH decline. Animals with borderline pH (5.9–6.1) end up being very tough [34]. Thus, minimising stress during transportation and slaughter should minimise meat tenderness depreciation. The transcriptomic response to emotional and physical stress before slaughter in two muscles and its relationships to meat quality was examined in cows. The transcriptomic evaluation showed a muscle-specific response to stress. It was characterised by the overexpression of 25 transcriptional modules in the stress-responsive genes of which 9 were common between muscles. The number of transcripts correlated with tenderness was significantly higher than expected by chance in the muscles of stressed cows. Positive correlations were detected between expression of muscle-specific genes (including genes relative to oxidoreduction, oxidative phosphorylation, and activity of the TCA cycle) and tenderness in the stressed animals [35]. The results indicated that mechanisms of tenderness are not similar in the muscles of stressed animals as compared to minimally stressed animals. In particular, the negative effects of stress were limited in cows showing a relatively high expression of genes involved in oxidative metabolic activity.

### 3.2. Complex Relationships of Biomarkers to Tenderness

Proteomic analysis highlighted that, for fast glycolytic muscle type, the more glycolytic the metabolism the more tender the beef cut and, for slow oxidative muscle, the more oxidative the metabolism the more tender the cut. Consequently, some biomarkers involved in the associated biological pathways will have relationships with tenderness dependent of the type of muscle. Our results combined with data from the literature clearly demonstrate that in glycolytic muscles (e.g., the ST) in breeds characterised by glycolytic muscle properties (e.g., the French Charolais, Limousin, Blond d'Aquitaine breeds) several proteins of the glycolytic metabolism (including PGM, LDH-B, triose-phosphate isomerase, glyceraldehyde-3-phosphate dehydrogenase, and ENO3) are positively correlated with tenderness. Conversely, in oxidative muscles such as (e.g., the LT) in breeds characterised by oxidative muscle properties (e.g., Aberdeen Angus) the proteins of the glycolytic metabolism are negatively correlated with tenderness, whereas several oxidative enzymes such as succinate dehydrogenase are positively correlated with tenderness [19, 21, 36]. Similar results are observed in French beef breeds for contractile proteins including the MYHC-IIx (fast glycolytic) or troponin T fast isoforms found to be positively associated with tenderness in the ST and negatively in the LT. In Aberdeen Angus, inverse observations were made with a negative relationship with tenderness in ST and a positive relationship in LT [36].

An inverse relationship between tenderness and proteins from the small Hsp family (Hsp20, Hsp27, and CRYAB) according to muscle type and breed has also been highlighted in the LT [36]. In French beef breeds these proteins are negatively related to tenderness in the ST muscle and positively in the LT muscle. The opposite was observed in Aberdeen Angus cattle or in breeds characterised by oxidative muscle properties such as dairy breeds [36, 37] and Picard (personal communication). This could be explained by differences in small Hsps abundance according to muscle types, as small Hsps are known to be more highly expressed in slow oxidative muscles [11]. Several studies showed a negative relationship between *µ*-calpain and tenderness in the LT but a positive one in the ST. An association was detected between small Hsps and *µ*-calpain [30]. It was proposed that CRYAB could act as a competitive inhibitor of *µ*-calpain activity against myofibrillar proteins [11]. This is consistent with the detection of inverse relationships of these proteins and tenderness according to muscle type.

However, for several biomarkers the relationships with tenderness are independent of the muscle type. For example, Hsp70-1B was found to be negatively related to tenderness in both the ST and the LT muscles from two French beef breeds and from Aberdeen Angus [36]. The *α*-actin was identified as a positive biomarker of tenderness by several authors, in different muscles and several breeds [19, 21, 38]. Other structural proteins including CapZ-*β* and desmin were also proposed as positive biomarkers of tenderness by several authors (for review [39]). The differences according to muscle type are in accordance with the two distinct molecular networks related to tenderness proposed by Guillemin et al. [28] for the two muscles.

## 4. From Biomarkers to Molecular Mechanisms of Meat Quality

Integration of biomarker data is a promising strategy to decipher the molecular mechanisms and the biological networks controlling beef quality (see Box 1). So far genomic experiments provided catalogues of genes or proteins. Data from many groups indicate that the level of gene expression* per se* and more precisely the combination of individual gene expression, rather than expression of a master gene, are responsible for phenotype variability (e.g., beef tenderness or marbling).

### 4.1. Computational Biology

Mining the information available from genomic experiments with sophisticated bioinformatics is helpful to depict mechanisms by giving insight into functional pathways and may help in seeking new candidate biomarkers. Indeed, computational biology has allowed detecting a complex interplay of genes/proteins responding to intrinsic and environmental factors for sensory attributes [28, 40]. The networks constructed using bioinformatics tools [28] or based on correlations [30] revealed some differences and similarities in the role of some proteins in tenderness, between the ST and the LT muscles. In the molecular network of tenderness constructed by Guillemin et al. [28], HSPB1 (Hsp27), HSPB6 (Hsp20), and CRYAB had a central role in the LT muscle. In the ST muscle, the most important proteins were DNAJA1 (Hsp40), HSPA8 (Hsp70-8), and HSPA1A (Hsp70-1A). According to protein functions, we could hypothesise that chaperone, antiapoptotic, and antistress functions are more active in the oxidative muscle LT. This is consistent with previous results [11] showing a higher abundance of stress-related proteins (HSPB1, HSPB6, CRYAB, DNAJA1, HSPA8, and HSPA1A) in LT than in ST muscle in young Charolais bulls. Gagaoua et al. [41] examined correlation networks between proteomic markers of tenderness in two muscles of three breeds (Aberdeen Angus, Blond d'Aquitaine, and Limousin). Several robust relationships were found between proteins belonging to similar or different biological pathways. Particularly, DJ-1 and PRDX6 were correlated with Hsp20 and *μ*-calpain, respectively. Proteins with cell protective functions, particularly antioxidative proteins and Hsps, are likely to play key roles. However, the mechanisms underlying tenderness according to muscle, breed, and gender still need to be elucidated.

### 4.2. Model Animals to Deepen Understanding of Beef Quality

Relevant information in beef is often lacking due to the incompleteness of annotation of the genome. Alternative strategies are to mine genome-wide sets of data from international databases (*in silico* approach) thanks to online and interactive workflows and databases [42, 43] or to use model species (*in vivo* and* in vitro* approaches). This is useful to reveal gene networks involved in the construction of the quality phenotype, for example, regarding the development of muscle and adipose tissues that determine the lean to fat ratio [44]. The knowledge gained from the studies in nonruminant and ruminant species can foster our understanding of biological mechanisms. For example, recent studies including ours have identified the heat shock protein Hsp27 as a beef tenderness biomarker [9, 14, 16, 19, 45] with differential expression in the muscle of animals giving high versus low meat tenderness. The protein is present as a hub node in a molecular network of biomarkers of tenderness [28, 45]. However, the relationships of Hsp27 with tenderness are not fully understood especially because of the complexity of its relationships to tenderness as illustrated above. Our hypothesis was that it may play a crucial role for the conversion of muscle into meat. In an attempt to depict the contribution of Hsp27 to tenderness, we engineered an Hsp27 null-mouse. We observed a muscle type specific alteration of the molecular phenotype in relation to apoptosis, Hsp status, and antioxidant status in an oxidative muscle. Changes in the Hsp status and calcium homeostasis were recorded in a glycolytic muscle. Electron microscopy revealed ultrastructural abnormalities in the myofibrillar structure of the mutant mouse [46]. Thus Hsp27 could directly impact the organisation of muscle cytoskeleton and contribute to tenderness at the molecular and ultrastructural levels, especially in oxidative muscles.

### 4.3. Biomarkers Turned to Beef Quality Diagnostic Tools

**Figure 3:**
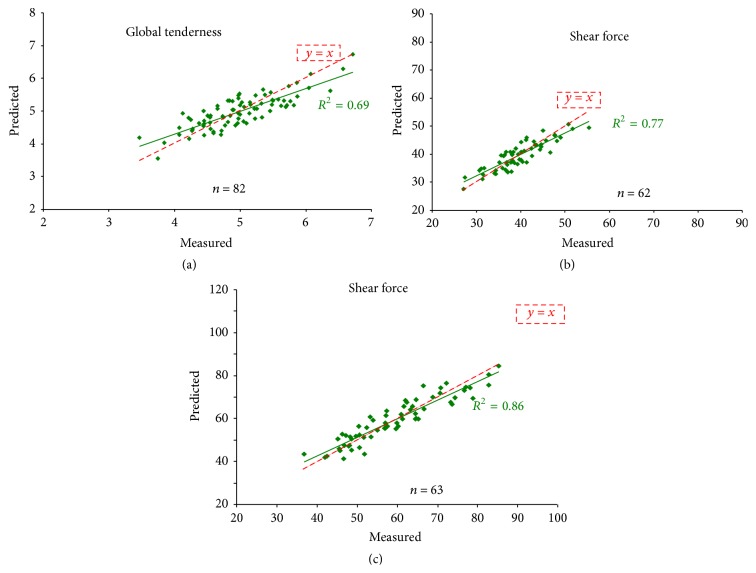
Equation of prediction using 24 protein biomarkers of (a) global tenderness evaluated by sensory analysis with trained panellists (scores from 1 to 10) on* longissimus thoracis* muscle, (b) shear force measured by Warner-Bratzler test (N/cm^2^) on* longissimus thoracis* muscle, and (c) shear force measured by Warner-Bratzler test (N/cm^2^) on* semitendinosus *muscle, in Charolais young bulls.

To address beef industry expectations, the way forward is to develop tools for marker quantification and meat prediction. The tests should be informative of the desired phenotype, for example, based on a combination of markers for sensory traits, and enable the evaluation of the beef potential of many head of cattle simultaneously. Developing a diagnostic test also requires that the technology for performing the assay should be affordable and readily automatable. First generation marker-based tools have been developed so far with private companies and stakeholders: a DNA chip dedicated to transcriptomics [16] and a dot-blot-array for protein profiling [47]. Up to now, they have been mostly used for research purposes. Converting data into knowledge of benefit to the livestock industry will soon not be a limitation anymore. Since proteins are easy to handle and to target, protein-based assays are preferable. We screened and selected the antibodies that worked best for biomarker evaluation. The dot-blot assay has been used to analyse high numbers of samples and to compute equations of prediction [36]. For example, on young Charolais bulls, Guillemin et al. [47] proposed equations of prediction based on the relative abundance of 24 protein biomarkers of tenderness (Figure 3). In the LT muscle, the prediction was higher for Warner-Bratzler shear force than for tenderness evaluated by sensory analysis. The prediction of shear force of ST muscle was better than that of LT, for the same animals. To date a technique based on immunodetection for large-scale analysis of a high number of proteins is under development. It will allow construction of a robust assay for high-throughput marker quantification for the beef sector. The next step will be to develop an algorithm to compute prediction values based on equations of prediction.

As illustrated above, a limitation of the expression marker-based strategy is that the markers are often specific of muscle type, animal type, livestock practices, or environmental conditions. So specific adaptations of the predictive tests of beef quality according to bovine breed, rearing practices, animal type, and beef cut will be necessary. The source of the sample will be another factor for the ease of test utilisation. The development of minimally invasive markers, especially plasma markers, is therefore promising for on-farm or in-abattoir use. By taking advantage of the progress in plasma proteomics for disease application in humans, we are using a proteome approach in order to identify minimally invasive biomarkers for beef performance and meat quality [48]. The plasma biomarkers would likely be part of the muscle secretome. As a proof of concept that examining the muscle secretome should help reveal plasma biomarkers, we identified* in silico *342 bovine proteins containing a signal of secretion in their sequence from a data set of 524 human muscle proteins [49, 50]. The list of proteins belonging to this bovine virtual muscular secretome was compared to a preliminary list of 316 proteins belonging to the bovine plasma proteome [48]. Fifty-two proteins secreted by the muscle and present in the plasma were revealed: they are mainly regulators of apoptosis, endopeptidase activity, and cell adhesion, antioxidant, and extracellular matrix interactors (Cassar-Malek and Tournayre, unpublished data). A current bioinformatics study is examining more deeply the composition of the plasma proteome in muscle secreted proteins.

Whether from muscle or plasma, the integration of biomarkers in detection tests should help early phenotyping of beef cattle. Their use in farms or in slaughterhouses shall ensure proper breeding programmes or management practices of the desirable live animals and their carcasses and the release of regular quality meat. By enabling early phenotyping in live animals, they will no doubt help in decisions regarding the way the animals should be farmed. Lastly the biomarkers can be further implemented in genetic tools for polymorphic genes or genes located in QTL regions for the phenotype that can be proposed as candidate genes and positional expression to explain the effects of QTL as shown in pigs or chicken (for a review see [8]).

## 5. Conclusion

**Figure 4:**
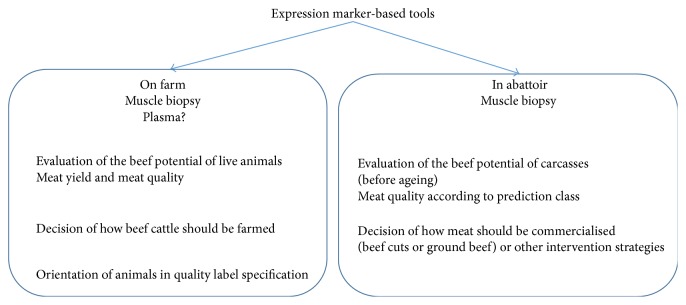
Expression marker-based tools for the management of sensory quality in the beef production chain. The application of the tools to the live animals or to their carcasses online will be for phenotyping and prediction purposes.

This paper reviewed the progress in identifying key genes and proteins to unravel the biology of beef quality. Expression marker-based strategy has allowed us to move forward in the understanding of beef sensory attributes. We will soon deliver effective molecular tools for the management of sensory quality in the beef production chain and the marketing of consistent quality meat (Figure 4). Its application to live animals at the farm or in testing stations will help phenotype animals in order to adapt breeding systems to fulfil expected quality outcomes.

## Figures and Tables

**Box 1:**
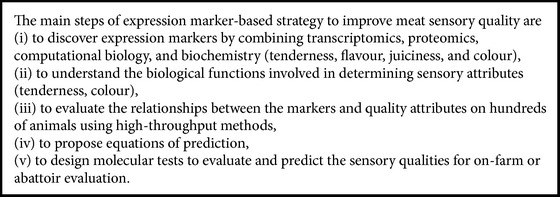


**Table 1:** List of proteins and gene identified to be associated with beef tenderness by transcriptomic and/or proteomic analyses according to [9, 17].

Protein name	Gene name	Function
Actin-*β*	ACTB	Cellular structure
Acyl-CoA desaturase	SCD	Lipid metabolism
Acyl-coenzyme A thioesterase 2	ACOT2	Lipid metabolism
ATP synthase chain B	ATP5B	Lipid metabolism
Calpastatin	CAST	Proteolysis
CapZ*β*	CAPZB	Cellular structure
Caspase 3	CASP3	Apoptosis
Caspase 8	CASP8	Apoptosis
Cis-Peroxiredoxin	PRDX6	Oxidative stress
*α* chain B crystallin	CRYAB	Cellular stress
Desmin	DES	Cellular structure
Diacylglycerol O-acyltransferase	DGAT2	Lipid metabolism
DJ-1	PARK7	Oxidative stress/androgen regulation
Enolase 1	ENO1	Energy metabolism
Enolase 3	ENO3	Energy metabolism
Hsp20	HSPB6	Cellular stress
Hsp27	HSPB1	Cellular stress
Hsp40	DNAJA1	Cellular stress
Hsp60	HSPD1	Cellular stress
Hsp70-1A/B	HSPA1B	Cellular stress
Hsp70-8	HSPA8	Cellular stress
Hsp70-Grp75	HSPA9	Cellular stress
Lactate dehydrogenase chain B	LDHB	Energy metabolism
Malate dehydrogenase 1 (cytoplasmic)	MDH1	Energy metabolism
Malate dehydrogenase 2 (mitochondrial)	MDH2	Energy metabolism
m-calpain	CAPN2	Proteolysis
*μ*-calpain	CAPN1	Proteolysis
Myosin binding protein H	MYPBH	Cellular structure/contraction
Myosin heavy chain I (slow)	MYH1	Cellular structure/contraction
Myosin heavy chain II (fast)	MYH2	Cellular structure/contraction
Myosin light chain 1F	MYL1	Cellular structure/contraction
Myosin regulatory light chain 2	MLC2	Cellular structure/contraction
NADH	NADH	Energy metabolism
Phosphoglucomutase	PGM1	Energy metabolism
S100-A1	S100-A1	Contraction/signaling
Superoxide dismutase Cu/Zn	SOD1	Oxidative stress
Superoxide dismutase (mitochondrial)	SOD3	Oxidative stress
Triose phosphate isomerase	TPI	Energy metabolism
Tropomyosin 3	TPM3	Cellular structure/contraction
Troponin T1	TNNT1	Cellular structure/contraction
Troponin T3	TNNT3	Cellular structure/contraction

**Table 2:** Expression markers of tenderness, juiciness, and flavour in the *longissimus thoracis* of young Charolais bulls (adapted from [9]).

Symbol	Gene name	Tenderness	Juiciness	Flavour
Upregulated transcripts				
C:6970	Homo sapiens chromosome 5 clone CTD-2151N11		^*∗∗*^	^*∗∗*^
CACNA1C	Calcium channel, voltage-dependent, L type, R 1C subunit		^*∗∗*^	^*∗∗*^
*Cbr2*	*Carbonyl reductase 2*	^*∗*^	^*∗*^	^*∗∗*^
CCNA1	Cyclin A1			
CGREF1	Cell growth regulator with EF-hand domain 1		^*∗*^	^*∗∗*^
CPT1B	Carnitine palmitoyltransferase 1B (muscle)		^*∗*^	^*∗*^
*FLJ12193*	*Hypothetical protein FLJ12193*	^*∗*^	^*∗∗*^	^*∗∗*^
Ireb2	Iron-responsive element binding protein 2		^*∗*^	^*∗*^
JMJD1B	Jumonji domain containing 1B		^*∗∗*^	^*∗∗*^
LAMA3	Laminin, R 3			^*∗∗∗*^
MPDZ	Multiple PDZ domain protein		^*∗∗*^	^*∗∗*^
NDUFB4	NADH dehydrogenase (ubiquinone) 1 f3 subcomplex, 4, 15 kDa		^*∗*^	^*∗*^
*Npm3*	*Nucleophosmin/nucleoplasmin, 3*	^*∗*^	^*∗*^	^*∗*^
OTOR	Otoraplin		^*∗∗*^	^*∗∗*^
PRKAG1	Protein kinase, AMP-activated, *γ* 1 non catalytic subunit	^*∗*^	^*∗∗*^	
PRRX2	Paired related homeobox 2		^*∗*^	^*∗∗*^
*TRIM55*	*Tripartite motif-containing 55*	^*∗*^	^*∗∗*^	^*∗∗*^

Downregulated transcripts				
CSRP3	Cysteine- and glycine-rich protein 3 (cardiac LIM protein)	−	−^*∗*^	−^*∗*^
DNAJA1	DnaJ (Hsp40) homologue, subfamily A, member 1	−^*∗∗*^	−	−

Only differentially expressed transcripts of which the abundance was correlated with a quality attribute (asterisk) are presented in the table. ^*∗∗ ***∗**^ *P* < 0.001; ^*∗∗*^ *P* < 0.01; ^*∗*^ *P* < 0.05; (−): negative correlation.
